# Indirect evolution of social fitness inequalities and facultative social exploitation

**DOI:** 10.1098/rspb.2018.0054

**Published:** 2018-03-28

**Authors:** Ramith R. Nair, Francesca Fiegna, Gregory J. Velicer

**Affiliations:** 1 Institute for Integrative Biology, ETH Zürich, Zürich, Switzerland; 2 Institute of Biology, Leiden University, Leiden, The Netherlands

**Keywords:** evo-devo, facultative cheating, fruiting bodies, multicellular development, non-adaptive evolution

## Abstract

Microbial genotypes with similarly high proficiency at a cooperative behaviour in genetically pure groups often exhibit fitness inequalities caused by social interaction in mixed groups. Winning competitors in this scenario have been referred to as ‘cheaters’ in some studies. Such interaction-specific fitness inequalities, as well as social exploitation (in which interaction between genotypes increases absolute fitness), might evolve due to selection for competitiveness at the focal behaviour or might arise non-adaptively due to pleiotropy, hitchhiking or genetic drift. The bacterium *Myxococcus xanthus* sporulates during cooperative development of multicellular fruiting bodies. Using *M. xanthus* lineages that underwent experimental evolution in allopatry without selection on sporulation, we demonstrate that interaction-specific fitness inequalities and facultative social exploitation during development readily evolved indirectly among descendant lineages. Fitness inequalities between evolved genotypes were not caused by divergence in developmental speed, as faster-developing strains were not over-represented among competition winners. In competitions between ancestors and several evolved strains, all evolved genotypes produced more spores than the ancestors, including losers of evolved-versus-evolved competitions, indicating that adaptation in non-developmental contexts pleiotropically increased competitiveness for spore production. Overall, our results suggest that fitness inequalities caused by social interaction during cooperative processes may often evolve non-adaptively in natural populations.

## 1. Background

Cooperation is prevalent across all major branches of life, including among both microbes [1–4] and multicellular animals [5–7]. In microbes, although cooperation may often occur between genetically identical cells [1,8–10], organisms proficient at cooperation also frequently interact in genetically heterogeneous social groups [4,11–13]. However, the evolutionary causes of divergence in social fitness between conspecific natural isolates that interact during cooperative processes are often unclear [13–17].

Here, we focus on social competition during microbial fruiting body development. Some microbes, including the prokaryote *Myxococcus xanthus* and the eukaryote *Dictyostelium discoideum,* respond to starvation by aggregating into groups composed of thousands of individuals that collectively construct multicellular fruiting bodies. Within fruiting bodies, subpopulations differentiate into spores that can survive stresses such as heat and starvation [18,19]. In both species, some cells die during development and this may benefit surviving spores [20,21].

During fruiting-body formation, genetically distinct individuals may co-aggregate into the same fruiting body [4,12,22,23]. When this occurs, differences in spore production by competitors are common [4,23]. Such differences in chimeric groups might result from (i) intrinsic (or ‘fixed’ [4]) differences between genotypes at sporulation in pure groups, (ii) responsive interactions between genotypes or (iii) a combination of fixed differences and social responses (electronic supplementary material, table S1) [13,14,23]. Here, we refer to relative-fitness inequalities that are specifically caused by social interactions between genotypes as ‘interaction-specific fitness inequalities' (or ISFIs) (see electronic supplementary material for definitions of this and other terms).

Competition outcomes in which one genotype produces more spores than another during fruiting body development in mixed groups due to ISFIs have been referred to as ‘cheating’ (or ‘facultative cheating’) in some *Dictyostelium* studies [4,14,17,24–29]. In this usage, ‘cheating’ refers to the superiority of a winning competitor at spore production in mixed groups, sometimes without reference to whether or not winning competitors increase their absolute level of spore production upon interacting with a losing competitor. In studies of *Myxococcus* development, ‘cheating’ has normally been applied to a fitness scenario in which one strain that outcompetes another in mixed developmental groups exhibits both (i) lower spore production in pure culture than does the competitor that loses in mixed groups and (ii) increased absolute spore production in mixed groups (relative to pure groups) due to social interaction with the losing competitor [13,23,30,31]. In keeping with previous practice, competitive superiority during development that is not known to involve social exploitation (defined as an interaction-specific increase in absolute spore production) is not referred to as cheating here. For competitions between two strains with similar pure-culture sporulation in which one strain does increase its absolute sporulation upon interacting with the other genotype, we refer to this as ‘facultative social exploitation’ [23,31], because the exploiting strain is not dependent on such exploitation to survive or produce large numbers of spores. (This scenario of fitness relationships has also been labelled ‘self-promotion’ in some *Dictyostelium* studies [14,32,33].)

ISFIs among microbial natural isolates are commonly discussed in the context of the potential for selection on developmental competitiveness in chimeric groups [4,14–17,23], but the evolutionary forces that generate such fitness asymmetries are generally unclear. Consonant with Gould & Lewontin's [34] famous reminder that non-adaptive explanations for the origins of organismal features should be adequately considered, it has been suggested that traits causing ISFIs during fruiting-body development might evolve indirectly [13,14,16,33], for example as by-products of differential local adaptation by allopatric lineages [13,16] or by genetic drift [13,16,30,33]. While previous studies with *Myxococcus* have shown that other social-interaction phenotypes such as quantitatively extreme forms of social cheating [35,36] and novel forms of kin discrimination [37] can readily evolve unselected, the potential for ISFIs and social exploitation between cooperation-proficient microbes to evolve non-adaptively remains largely unexamined.

One means by which ISFIs in microbial fruiting-body development might evolve is divergence in the rates at which distinct strains undergo fruiting-body development in pure groups. Kraemer *et al*. [38] showed that distinct natural isolates of *M. xanthus* vary greatly in developmental speed and suggested that such differences might generate fitness inequalities in chimeric groups. Smith *et al.* [16] presented a model predicting that slow development should reduce competitiveness if fast development allows disproportionate access to developmental signals. Experiments with *D. discoideum* have generated results of variable consistency with this hypothesis [39,40], but to our knowledge this hypothesis has not been tested directly.

**Figure 1.**
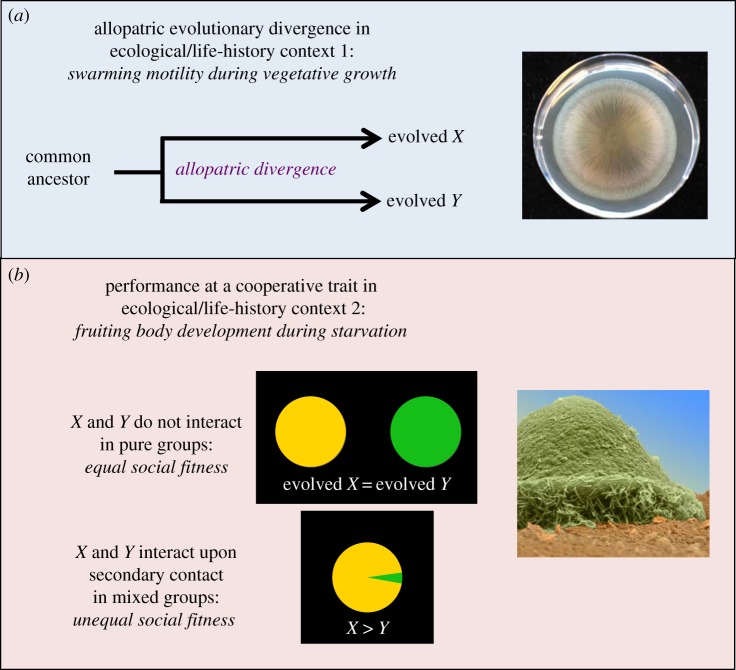
Indirect evolution of interaction-specific developmental fitness inequalities. (*a*) Allopatric evolution of distinct *M. xanthus* lineages during selection for increased fitness at the leading edge of vegetatively growing colonies expanding by gliding motility. In our actual experiments, all lineages derived from a very recent common ancestor (strain GJV1). However, some evolved lineages differ only by mutations that accumulated during experimental evolution and a genetic marker whereas others also differ at an engineered mutation in a motility gene introduced prior to the start of experimental evolution. Some evolved lineages underwent independent evolution in the same environment whereas others evolved on plates that varied in nutrient source and/or agar concentration. (See main text and electronic supplementary material, tables S2 and S3 for details.) (*b*) Transfer of populations that evolved in one ecological context to a very different context, namely starvation conditions that induce multicellular fruiting body development. In this study, strain pairs that retained similarly high levels of spore production in pure culture development assays (represented by equal-size yellow and green circles) were identified and then examined for inequalities in spore production caused by social interaction upon secondary contact (i.e. forced experimental mixing at an initial 1:1 ratio) during development. The electron-microscopy image of an *M. xanthus* fruiting body was prepared by Jürgen Berger (Max-Planck Institute for Developmental Biology) and Supriya Kadam. (Online version in colour.)

We jointly tested both the hypothesis that ISFIs and facultative social exploitation can readily evolve non-adaptively as unselected by-products of selection on other traits (or random processes) and the hypothesis that speedy development exhibited in pure culture tends to be competitively advantageous in mixed groups (electronic supplementary material, table S1). We first screened more than 100 experimentally evolved populations of *M. xanthus* for (i) evolutionary retention of developmental proficiency (i.e. the ability to form visible fruiting bodies with a high level of spore production) and (ii) indirect evolutionary divergence in rates of fruiting body development*.* These distinct population lineages had undergone selection in allopatry for increased competitiveness at the leading edge of vegetatively growing colonies that were actively swarming across agar surfaces under a variety of selective environments (figure 1) [37,41]. Importantly, these populations were not subjected to starvation-induced fruiting-body development, so any evolutionary changes in development-specific traits originated non-adaptively with respect to their effects on competitiveness during fruiting body development and sporulation.

Among populations that indirectly diverged in developmental speed, we identified pairs of evolved clones that exhibited similar levels of sporulation in pure culture. Using these clones, we paired fast- and slow-developing competitors to test for fitness asymmetries (electronic supplementary material, figure S1) and for any correlation between fitness and developmental speed. We further tested for indirect evolution of social exploitation by cooperation-proficient genotypes, namely positive effects of interaction between social competitors on the absolute fitness of the winning competitor. Finally, for a subset of evolved strains that all descended from the same pair of reciprocally marked ancestors, we tested whether they had changed in developmental competitiveness relative to their ancestors after evolution in non-developmental contexts and whether any such patterns of indirect evolutionary change predicted the outcomes of developmental competitions between evolved genotypes.

## 2. Material and methods

### (a) Strains and swarming-rate assays

The strains examined here were all experimentally evolved from six closely related ancestors that differed only in motility genotype and antibiotic-resistance marker state (electronic supplementary material, table S2) [37]. *Myxococcus xanthus* swarms on agar media using two distinct motility systems, ‘A-motility’ and ‘S-motility’ that involve different sets of genes [42]. Both motility systems were fully functional in two of our ancestors (GJV1 and GJV2), whereas the other four ancestors were defective at either A-motility (GJV3 and GJV5) or S-motility (GJV4 and GJV6) due to defined deletions in genes essential to either system (*cglB* and *pilA*, respectively). One ancestor from each genetic background was rifampicin sensitive (GJV1, GJV3 and GJV4) while the other was rifampicin resistant (GJV2, GJV5 and GJV6).

Details of the evolution experiments were described by Rendueles *et al.* [37] and Rendueles & Velicer [41]. Briefly, multiple replicate populations from each ancestor were allowed to swarm radially outward on different types of solidified agar media over two-week intervals (electronic supplementary material, table S3). At the end of each two-week cycle, an approximately 3 × 5 mm agar sample from the outermost point on the swarm perimeter was harvested and transferred to the centre of a fresh plate (or from a random location in the case of circular swarms). For purposes of this study, the most important features of the experimental-evolution design were that populations evolved independently of one another and were not under selection for increased fitness during fruiting body development. For this study, all population samples and clones were isolated after 18 two-week cycles of selection.

Swarming-rate assays were performed with evolved and ancestral strains in the relevant selective environments in which experimental evolution was conducted. Plates were prepared and rate assays were conducted as described in [37,41].

### (b) Screen for fruiting-body formation and developmental speed

For all experiments, cultures were inoculated from frozen stocks into 8 ml CTT liquid media (10 mM Tris pH 8.0, 8 mM MgSO_4_, 10 g l^−1^ casitone, 1 mM KPO_4_ (KH_2_PO_4_ + K_2_HPO_4_)) [43] in 50 ml conical flasks, which were incubated at 32°C with constant shaking at 300 r.p.m. until cultures reached mid-exponential phase (OD_600_ 0.5–1.0). To test whether experimentally evolved populations retained the ability to form fruiting bodies, liquid cultures of 136 evolved populations were centrifuged at 5000 r.p.m. for 15 min and resuspended in clone fruiting (CF) buffer (10 mM Tris pH 8.0, 1 mM KH_2_PO_4_, 8 mM MgSO_4_, 0.02 mg ml^−1^ (NH_4_)_2_SO_4_, 2 mg ml^−1^ citrate, 1 mg ml^−1^ pyruvate) [43] at a density of approximately 5 × 10^9^ cells ml^−1^. Cell densities of exponential-phase cultures were estimated by measuring optical density with a TECAN GENios™ microplate reader. Ten microlitre aliquots of re-suspended cultures were spotted onto CF nutrient-agar plates (CF buffer with 0.015% casitone and 1.5% agar) and incubated at 32°C, 90% humidity for 8 days. Images of developing cultures were taken daily using a ProgRes^®^ C5 imaging system with a Leica Wild M8 stereomicroscope at 8X magnification to determine developmental speed. For use in subsequent competition experiments, individual clones were isolated from each population that shared the same developmental-speed and sporulation-level phenotypes as the whole population from which they were isolated.

### (c) Developmental competition assays

Developmental competitions were performed in a manner similar to those reported by Fiegna & Velicer [23] except that competitions were performed on CF medium lacking Casitone (hereafter ‘CF−’ medium). Casitone is the major carbon source in the medium and was omitted from competition experiments to reduce any potential differences in competitiveness for growth-substrate utilization. All strains were confirmed to exhibit similar developmental speeds and fruiting body morphologies on CF− medium as on CF medium. Mid-exponential growth phase cultures grown in CTT media were centrifuged and resuspended in CF buffer at 5 × 10^9^ cells ml^−1^ and 100 µl of the suspension was spotted in the middle of a CF− 1.5% agar plate for pure culture controls. Mixed competition cultures included two competing strains with opposite marker states (also each resuspended to 5 × 10^9^ cells ml^−1^) mixed at a 1 : 1 ratio and 100 µl of the mix was spotted on CF− 1.5% agar plate. Pure culture assays of each competing strain were performed simultaneously with competition assays and all competitions were performed in three independent replicates.

Developmental plates were incubated at 32°C, 90% rH for 5 days, after which cells were harvested from the agar surface with a sterile scalpel blade, transferred into 0.5 or 1 ml ddH_2_O and heated at 50°C for 2 h to select for viable spores. Samples were then sonicated using a microtip sonicator, serially diluted with ddH_2_O plated into 10 ml of molten sterile CTT soft-agar medium (0.5% agar at 50°C) and incubated at 32°C, 90% rH for 7–10 days before colonies were counted. For all mixed competitions, samples were plated into both plain CTT soft agar and agar containing rifampicin (5 µg ml^−1^) to allow subtractive calculation of rifampicin-sensitive colony numbers.

### (d) Parameters

Spore count data were analysed as per Fiegna & Velicer [35]. Parameter calculations are also described in the electronic supplementary material.

All statistics were performed using open-source R statistical software [44] and RStudio (v. 1.1.383; Boston, MA).

## 3. Results

### (a) Intrinsic developmental proficiency and speed diverged indirectly

#### (i) Developmental proficiency

We first tested whether evolved populations retained the ability to form fruiting bodies and found that fewer than half (59/136) formed visually distinct fruiting bodies on CF− agar (electronic supplementary material, table S4). This result indicates that, like adaptation in high-nutrient liquid culture [45], adaptation by motile populations growing in high-nutrient structured habitats often causes mutations that reduce developmental proficiency to reach high frequency or fixation. Thus, extended exposure to high-nutrient growth conditions *per se* appears sufficient to relax selection for retention of developmental proficiency, irrespective of the degree of spatial structure in the environment. Among the 59 evolved populations that formed fruiting bodies, pure-culture spore production exhibited a largely continuous distribution spanning five orders of magnitude (approx. 10^3^ – approx. 10^8^; electronic supplementary material, figure S1).

#### (ii) Developmental speed

We initially screened the 59 fruiting-proficient populations for indirect evolutionary changes in developmental speed by documenting the earliest formation of opaque fruiting bodies (across 24-h intervals), which ranged between 1 and 5 days after the onset of starvation (e.g. electronic supplementary material, figure S2). All ancestral genotypes first showed mature dark fruiting bodies after 2 days except GJV6 (the rifampicin-resistant variant of GJV4), which never formed fully opaque fruiting bodies within 5 days. Of the evolved populations, 18 indirectly evolved a faster rate of fruiting-body development than their ancestor, 26 evolved slower development and 15 remained unchanged, at least at the resolution of 24-h intervals (electronic supplementary material, table S4).

### (b) Relative fitness in chimeric groups during development diverged indirectly

For the purpose of pairing strains for developmental competitions, populations that initially formed fruiting bodies within 1 or 2 days were classified as fast (31 populations) and those taking 3 or more days were classified as slow (28 populations) (electronic supplementary material, tables S4 and S5). There was no significant difference in the mean pure-culture spore production between fast- versus slow-developing populations (electronic supplementary material, figure S3, Wilcoxon two-sample test, *p* > 0.05). Because intrinsic spore production was highly variable across the evolved strains we examined, for competition experiments we paired only fast and slow clones that exhibited indistinguishable levels of spore production after 5 days in pure culture (electronic supplementary material, figure S1). Additionally, strains were only paired with a partner of the opposite rifampicin-resistance marker state and each strain was included in only one competition pairing so that all pairings represent independent divergences during experimental evolution. Competition pairings are detailed in electronic supplementary material, tables S5 and S6.

We first tested for simple differences in developmental competitiveness of paired evolved strains (which all ultimately descend from the same known laboratory ancestor GJV1), irrespective of the degree to which such differences are caused by mutations that accumulated during experimental evolution [37,41], by intentional deletion of either of the motility genes *cglB* or *pilA* from some of the proximate ancestors of the experimental lines or by ancestral rifampicin-resistance mutations present in half of all competitors. Importantly, none of the mutations that distinguish competitors (whether intentionally introduced ancestral motility or marker mutations or mutations that accumulated during experimental evolution) are present due to imposed selection for developmental competitiveness.

**Figure 2.**
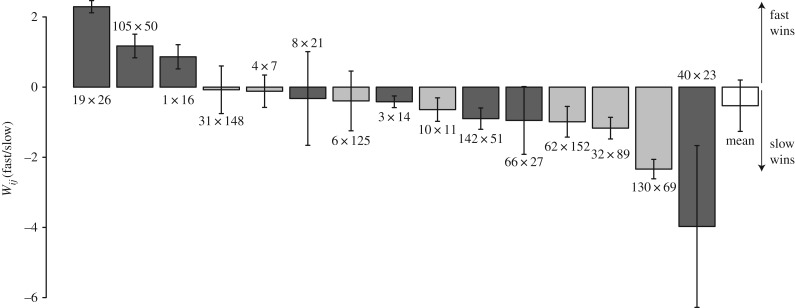
Relative fitness in mixed-group developmental competitions diverged indirectly. For each pair of strain identifiers, the strain listed on the left forms fruiting bodies more rapidly than the strain listed on the right in separate pure cultures. *W_ij_* estimates reflect the relative fitness of the faster-developing competitor in comparison to its slower-developing paired competitor in mixed competitions initiated at a 1 : 1 ratio. Positive values indicate that the fast-developing strain won the respective competition and negative values the opposite. Lighter bars denote competitions between strains with identical ancestral motility genotypes. Error bars represent 95% confidence intervals.

Across all pairs of evolved competitors, relative fitness during mixed developmental competitions diverged greatly (figure 2), with the winning strains producing more than 10-fold more viable spores on average than their paired competitors (average *W_ij_* of winning strain = ∼1.11, *p =* 0.001 for difference from 0, one-sample *t* test, d.f. *=* 14). Thus, because paired competitors had very similar performance at spore production in pure culture, genetic divergence that was non-adaptive with respective to a focal social trait (here competitiveness in chimeric fruiting body development) generated large fitness asymmetries specifically caused by interaction between paired competitors during the focal social process.

A large proportion of interaction-specific fitness divergence revealed by the analysis above is due to genetic divergence specific to experimental evolution rather than to defined motility mutations or rifampicin-resistance mutations in the proximate ancestors of the evolution experiment. First, a linear mixed-effect model with evolved-strain-pair identity as a fixed effect and corresponding ancestor-pair-identity as a random effect revealed that evolved-strain pair identity contributes significantly to the variance in *W_ij_* (*F*_14,24_ = 4.79, *p* < 0.001). Further, we subtracted estimates of developmental-competition *W_ij_* values between proximate experimental ancestors (e.g. GJV1 versus GJV2) (electronic supplementary material, figure S4) from the *W_ij_* values for respective evolved-competitor pairings to generate estimates of the parameter Δ*W_ij_*, which represents fitness divergence specific to experimental evolution (electronic supplementary material, figure S5). The average value of *ΔW_ij_* for winning strains across all competition pairs was 0.92, which is only slightly lower than the average value of *W_ij_* and is significantly greater than 0 (*p =* 0.002 for difference from 0, one-sample *t* test, d.f. *=* 14).

### (c) Fitness ranks of evolved competitors are not predicted by fixed variation in developmental speed

The hypothesis that being intrinsically faster at fruiting-body development than other genotypes generally increases competitiveness in mixed groups is not supported by our results. This hypothesis predicts that the average *W_ij_* of the faster-developing competitors should be significantly positive and that a significant majority of *W_ij_* values for fast strains should be positive as well. Contrary to these predictions, the overall mean of *W_ij_* estimates for the developmentally faster competitors was actually negative (−0.54), as were a majority (12/15) of individual estimates of fast-strain *W_ij_* values (figure 2). Qualitatively similar results are obtained by considering *ΔW_ij_* rather than *W_ij_* (electronic supplementary material, figure S4).

### (d) Developmental fitness ranks of evolved competitors are not predicted by evolutionary increases in swarming rate

Rendueles & Velicer [41] examined a subset of the lineages examined here for evolutionary changes in swarming rate in the environment in which they underwent experimental evolution. They found that after 40 two-week cycles of selection, a majority of evolved populations exhibited faster swarming than their ancestors, but a substantial minority did not and some were actually slower than their ancestors. We also tested for altered swarming rates among the evolved populations examined here after 18 cycles of selection in their experimental-evolution environment for evolved strain pairs that exhibited *W_ij_* values in developmental competitions that differed significantly from 0. Similar to the results of Rendueles & Velicer [41], a majority of populations increased in swarming rate while some showed no substantial difference from their ancestor and others were slower (electronic supplementary material, figure S6). Pertaining to developmental competitions, whether or not an evolved strain increased swarming rate in its experimental-evolution environment was not predictive of its fitness rank in developmental competitions. For three competition pairs, one competitor appears to have increased in swarming rate while the other did not, for four other pairs both competitors appear to have increased in swarming rate and for two pairs neither competitor exhibited faster swarming than its ancestor.

### (e) Facultative social exploitation evolved indirectly

**Figure 3.**
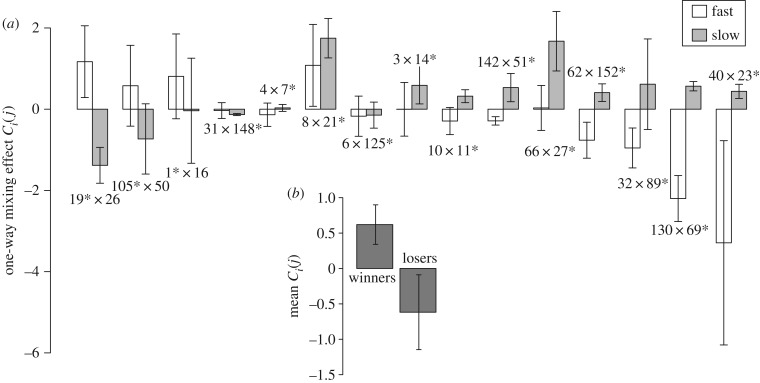
Evolved strains exhibit facultative social exploitation and antagonisms toward one another upon interaction during development. (*a*) Positive and negative *C_i_*(*j*) values signify increased or decreased absolute sporulation efficiency as a result of interacting with a competitor, respectively. Competition pairs are shown in the same order as in figure 2 with asterisks indicating strains with a positive *W_ij_* estimate for each competition. (*b*) Average *C_i_*(*j*) values of winning (*W_ij_*> 0) and losing (*W_ij_* < 0) competitors across all 15 competition pairings. Error bars represent 95% confidence intervals.

Evolutionary divergence in relative fitness during competitions in chimeric groups between strains that exhibit similar absolute social performance in pure culture might result from any of several combinations of changes in interaction-specific absolute performance. For example, both competitors might have indirectly evolved negative responses to mixing, but if one negative response is of greater magnitude than the other a difference in relative fitness will result (e.g. fig. 5 of [23]). Inversely, two strains might both evolve positive absolute responses to mixing but nonetheless have unequal relative fitness. Alternatively, fitness asymmetries might be generated by strains responding oppositely to mixing or by only one strain changing its sporulation level. To characterize responses of the absolute fitness of individual competitors to encounters with their respective paired competitor and how such responses may affect relative-fitness outcomes, we quantified the effects of mixing on absolute sporulation levels with estimates of the parameter *C_i_*(*j*) (see Material and methods) (figure 3*a*). On average across all competition pairs, the absolute fitness (i.e. sporulation efficiency) of those competitors with a positive *W_ij_* estimate (i.e. the winning competitors) was found to increase significantly (approx. fourfold) in response to interaction with their respective paired losing competitor (figure 3*b*, *p* = 0.0007 for difference from 0, one-sample *t* test, d.f. *=* 14). Multiple positive mixing effects were estimated to involve increases in spore production by the winning strains greater than 10-fold in magnitude (pairs 19 × 26, 8 × 21 and 66 × 27) relative to pure-culture levels.

By contrast, the losing strains (those with negative *W_ij_* estimates) decreased in absolute fitness from interaction with the respective winning competitors, on average (figure 3*b*). These results indicate that winning strains do not win merely by decreasing the absolute fitness of their competitors, but also because their own absolute fitness is often increased by interaction with those competitors. In other words, the winners socially exploited interaction with the losers for an absolute gain and did not merely suppress the losers for a relative advantage. Considering the independent evolutionary histories of competing strains in non-developmental selective contexts, it is clear that at least some strains indirectly evolved the ability to facultatively exploit competitors to which they were previously naive without having undergone any selection for developmental competitiveness *per se*.

### (f) Degree of evolutionary change in developmental competitiveness compared to ancestors predicts the relative fitness of evolved–evolved competitors qualitatively, but not always quantitatively

Developmental fitness inequalities between derived genotypes could evolve indirectly by a wide range of scenarios. In one major category of such scenarios, the relative fitness of any two evolved competitors might be closely predicted by the relative performance of those genotypes in mixed competition with their reciprocally marked ancestors. Alternatively, social epistasis (i.e. genotype-by-genotype interactions in a social context) between evolved and ancestral genotypes or between evolved genotypes may generate unpredictable competition outcomes.

To test between these alternatives, we performed further developmental competitions involving four evolved versus evolved pairings for which the evolved competitors share the same motility-genotype ancestor and for which one competitor produced significantly more spores than the other in mixed competitions (i.e. had a *W_ij_* value significantly different than zero, figure 2). These four pairs of evolved strains (11 × 10, 89 × 32, 152 × 62, 69 × 130) all descended from the wild-type motility genotype (A + S+) ancestors GJV1 or GJV2. For each pair, we performed four categories of competition experiments during development simultaneously: evolved versus evolved (aka Ev versus Ev), evolved versus reciprocal ancestor (for both evolved strains, aka Ev versus Anc) and ancestor versus ancestor (GJV1 versus GJV2).

**Figure 4.**
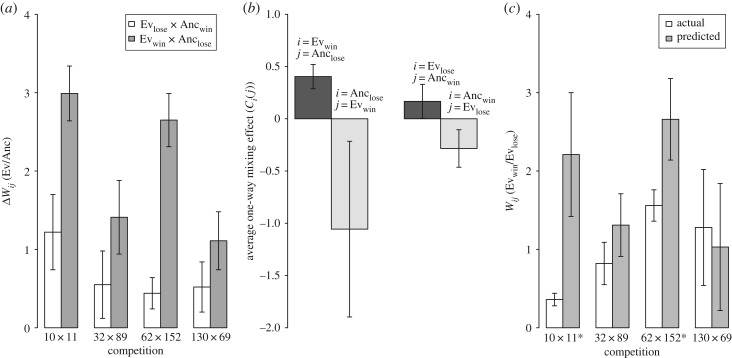
Evolution during vegetative growth and swarming increased competitiveness against ancestors during starvation-induced development, but to a greater extent among winners of evolved-versus-evolved competitions. (*a*) Each Δ*W_ij_* value represents the change in developmental competitiveness of the focal evolved strain relative to its reciprocal ancestor due to experimental evolution. The (small and non-significant) *W_ij_* value between the two ancestors (GJV1 versus GJV2) has been subtracted from the value obtained from the evolved-ancestor competition to yield the evolutionary change in developmental competitiveness (‘Ev_win_’ and ‘Ev_lose_’ refer to the winner and loser of an evolved–evolved competition and ‘Anc_win_’ and ‘Anc_lose_’ refer to the proximate ancestors of those evolved competitors, respectively). (*b*) Average one-way mixing effect *C_i_*(*j*) estimates for Ev_win_ and Anc_l*ose*_ competitors in Ev_win_–Anc_lose_ pairings and for Ev_lose_ and Anc_win_ competitors in Ev_lose_–Anc_win_ pairings. In all cases, the *C_i_*(*j*) value pertains to the strain category *i* when mixed with the strain category *j.* (*c*) Predicted and actual *W_ij_* values for the winning competitor of each evolved–evolved pair for mixed competitions during development. Predicted values are based on the values of evolved-ancestor competitions. Strains 11, 89, 152 and 69 were the winning competitors for these four pairs. Asterisks indicate significant differences between predicted and actual outcomes. Error bars represent 95% confidence intervals.

As in the first set of mixed competitions, the two ancestors exhibited no significant difference in spore production (average *W_ij_* of GJV1 = 0.22, *p >* 0.05 for difference from 0, one-sample *t* test, d.f. *=* 3). Factoring out this small, non-significant fitness difference estimate between GJV1 and GJV2, we found that all evolved populations appear to have increased greatly in developmental fitness against their reciprocally marked ancestor in mixed competitions (figure 4*a*), including the four strains that lost in evolved versus evolved competitions (10, 32, 62 and 130). These four losing strains collectively exhibited a significant advantage over their ancestors (grand mean *W_ij_* = 0.68, *p <* 0.05, one sample *t* test). For all four repeated evolved versus evolved competitions, the same evolved strain won as in our first set of competitions (figures 2, 4*a* and 4*c*).

For both evolved versus ancestor and evolved versus evolved competitions, higher developmental fitness was associated with social exploitation. Not only was the average one-way mixing-effect parameter *C_i_(j)* of evolved winners found to be significantly positive when they were competed against their reciprocal ancestors, but so was that of the evolved losers (figure 4*b*; electronic supplementary material, figure S7, grand mean *C_i_(j)* values of evolved winners and losers, respectively, are 0.404 (*p* = 6.03 × 10^−6^) and 0.269 (*p* = 0.0005), respectively, one sample *t* tests versus 0).

For all four focal evolved–evolved pairs, the evolved strain that won the respective Ev–Ev competition in our previous experiments (Ev_win_) showed a greater advantage over the ancestor than did the loser of that competition (Ev_lose_) (figure 4*a*). Thus, at least for these four competition sets, the relative degree of increase of developmental fitness of the two evolved strains over their ancestors was qualitatively predictive of the winner of Ev–Ev competitions. Quantitatively, using the results of the two Ev versus Anc competitions from each set (Ev_win_ versus Anc_lose_, Ev_lose_ versus Anc_win_) we calculated the expected developmental fitness advantage *W_ij_* of Ev_win_ over Ev_lose_ if there were no genotype-by-genotype fitness interactions specific to any of the relevant strain pairings (figure 4*c*). In two cases (Ev89_win_ versus Ev32_lose_ and Ev69_win_ versus Ev130_lose_), the magnitude of the actual Ev_win_ advantage did not differ significantly from the predicted *W_ij_* value (*p* > 0.35), whereas in the other two cases (Ev11_win_ versus Ev10_lose_ and Ev152_win_ versus Ev62_lose_) the actual *W_ij_* values were significantly smaller than predicted (figure 4*c*, *p =* 0.012 and 0.032, respectively, Bonferroni-corrected *t* tests).

## 4. Discussion

Our results show that fitness effects of social interaction between cooperation-proficient microbes that are often discussed in the context of selection for competitiveness during a focal social process can easily evolve as by-products of other processes. In particular, interaction-specific developmental fitness inequalities (figures 2 and 3) as well as facultative social exploitation during development (figure 3) are shown to readily evolve as by-products during adaptation in selective regimes lacking a developmental phase.

These findings inform interpretation of social fitness differences between interacting microbes with unknown selective histories, such as most natural isolates. Many biological traits evolve indirectly [46–51], including other striking social-interaction phenotypes among microbes. For example, some of most quantitatively extreme cases of microbial cheating yet reported (i.e. cheating by developmentally defective strains of *M. xanthus*) evolved indirectly [36]. Similarly, territorial kin discrimination among microbes [13,52,53] has also been shown to pervasively evolve in an indirect manner [37]. Collectively, these results suggest that by-product divergence of traits affecting developmental competitiveness is a highly plausible default hypothesis for explaining the origin of fitness asymmetries between natural isolates that are specific to chimeric development.

We also examined whether patterns of evolutionary change in developmental fitness relative to the ancestors of our evolved lineages might inform interpretation of how ISFIs between evolved competitors arose. For a set of four evolved-competitor pairings, all eight evolved populations (including all four Ev_lose_ strains) were found to have increased in developmental competitiveness relative to their ancestors in mixed competitions. However, in all four cases the Ev_win_ strains increased more than Ev_lose_ strains (figure 4*a*). These results suggest, first, that improved fitness at the leading edge of swarming colonies growing in nutrient-rich conditions tended to pleiotropically increase fitness in a radically different life-history/ecological context, namely starvation conditions that induce multicellular fruiting body development. Second, the relative degree of (indirect) evolutionary increase of developmental fitness of two evolved strains was in several cases qualitatively predictive of the winner of competitions between derived genotypes in that alternative selective context. Thus, even if two interacting evolved competitors both gained adaptations with positive pleiotropic effects on developmental fitness relative to their ancestor, differences in the relative magnitude of those effects appear to generate developmental ISFIs during evolved–evolved competitions.

The second finding above might suggest that interactions between evolved strains and their ancestors are highly predictive of interactions between evolved strains. However, we calculated the expected developmental fitness advantages of Ev_win_ over Ev_lose_ strains based on Ev versus Anc competitions under the assumption of quantitative transitivity of fitness relationships. In two cases, the magnitude of the winner's advantage was found to be much smaller than predicted, indicating strong G×G fitness interactions unique to the paired evolved genotypes in those cases (figure 4*c*). Thus, the magnitude of ISFIs generated indirectly by divergent evolution cannot be assumed to reflect differences in the magnitude of evolutionary fitness changes relative to ancestral states.

With respect to proximate mechanisms of developmental fitness asymmetry, it has been proposed that intrinsically fast-developing strains of *M. xanthus* might be expected to outcompete slow-developing strains [38] and that developmental speed differences are one means by which interaction-specific fitness asymmetries might evolve non-adaptively [16]. Our results from competitions between strains that differ substantially in their overall rate of multicellular morphological development but show similar spore productivity are inconsistent with this fixed developmental-speed hypothesis for the origin of by-product fitness inequalities. Alternative hypotheses (that are not mutually exclusive) include (i) socially responsive changes in the speed of development by one or both competitors induced by interaction with the other genotype, (ii) evolved differences in shorter-term temporal dynamics and levels of developmental signal production and/or sensitivity to competitor-produced signals that affect the final spore production of mixed strains differently and (iii) mechanisms akin to interference competition, such as production of a compound by one strain at a level that harms the other.

Working with the social amoeba *D. discoideum,* Parkinson *et al*. [33] showed that simple variation in the production of and responsiveness to a developmental signal (StlF) can generate interaction-specific alteration of relative spore production in developmental competition experiments without the need for complex behavioural adaptations for social exploitation. The authors also showed that such variation in signal production/responsiveness is common in natural populations and noted that this variation might be generated by evolutionary forces other than selection on developmental competitiveness [34]. Among our developmental competition pairings of *M. xanthus* strains that evolved independently in non-developmental contexts, a majority of estimates of responses to chimerism [*C_i_*(*j*)] by winning strains were positive, indicating or suggesting social exploitation (figure 3*a*), with the average response of winning competitors to interaction with their paired partners being significantly positive (figure 3*b*). Consistent with Parkinson *et al*. [33], these instances of indirectly evolved facultative exploitation may involve relatively simple increases in the sensitivity of some strains to intercellular signals triggering sporulation in mixed developmental competitions.

Inversely, most losing strains suffered absolute decreases in spore production due to interactions in chimeric groups (figure 3). Such suppression of sporulation by winning strains might be caused either by decreased sensitivity to sporulation signals by some strains or production of compounds (or levels of compounds) produced by winning strains that are toxic to losing strains. The latter hypothesis is plausible in light of pervasive instances of antagonism among natural isolates of *M. xanthus* [13,23,54] and the evolution of negative interactions between some of the lineages of the broader set of experimental-evolution populations examined here and their ancestors [41].

Regardless of what proximate mechanisms generate the positive and negative responses to interaction between developmentally proficient genotypes of *M. xanthus* documented here, our results make it clear that direct selection for those mechanisms is not required for them to evolve. In complex organisms (and even simple ones), pleiotropy is common [22,32,55–57] and in highly social organisms, non-adaptive pleiotropic side effects of adaptive mutations, as well as genetic linkage and drift of non-adaptive mutations, may often strongly determine the fitness effects of social interactions.

## Supplementary Material

Nair,Fiegna&Velicer_methods_tables_figures_ESM.pdf

## Data accessibility

Additional data have been provided as electronic supplementary material.
