# Supplementary material for: Indirect evolution of social fitness inequalities and facultative social exploitation
Source: Proc Biol Sci. 2018 Mar 28;285(1875):20180054. doi: 10.1098/rspb.2018.0054 (PMC5897644; doi:10.1098/rspb.2018.0054)
Supplement: Nair,Fiegna&Velicer_methods_tables_figures_ESM.pdf [file rspb20180054supp1.pdf]

Supplementary Material for:

Indirect evolution of social fitness inequalities and facultative social exploitation

Ramith R. Nair, Francesca Fiegna and Gregory J. Velicer

Proceedings of the Royal Society B

SUPPLEMENTARY METHODS

Parameters

The untransformed sporulation efficiency (D) of strain *i* in pure culture was defined as the frequency of cells surviving development as viable spores:

$$D_i = N_i(t_5) / N_i(t_0), \ldots\ldots 1$$

where N_i represents the viable population size of strain *i* before starvation (t_0) and after 5 d of starvation (t_5). The efficiency of *i* in a mixed competition assay with strain *j* is similarly given as

$$D_i(j) = N_i(j, t_5) / N_i(j, t_0) \ldots\ldots 2$$

The effect of mixing strains *i* and *j* on the sporulation efficiency of strain *i* is given as

$$C_i(j) = \log_{10}[D_i\ (j)] - \log(D_i) \ldots\ldots 3$$

Thus, a positive value of $C_i(j)$ indicates that strain *i* sporulates more efficiently in the presence of strain *j* than in clonal isolation, whereas a negative value indicates that strain *i* sporulation efficiency is decreased by coculture with strain *j*.

The relative sporulation efficiency of two strains in mixed competition is defined as the difference between the log-transformed actual sporulation efficiencies of two competing strains:

$$W_{ij} = \log_{10}(D_{i(j)}) - \log_{10}(D_{j\,(i)}) \ldots\ldots 4$$

The winner of a competition is reflected by the direction of W_{ij}. Positive and negative W_{ij} values indicate that *i* or *j* exhibited higher relative fitness than the other competitor, respectively.

The six closely related ancestors of the evolved strains (Table S1) exhibited some differences (mostly small) in pure-culture sporulation efficiency (Figure S1) and in relative spore production in mixed competition (Figure S4). To factor out the latter, we calculated the degree of evolutionary divergence in $W_{ij,}$ during experimental evolution, or ΔW_{ij} , where

$$\Delta W_{ij} = W_{ij} - W_{iaja}\,, \ldots\ldots 5$$

and '*a*' indicates the ancestor of the respective strain.

Semantics

Fitness: In this study, all measures of fitness are determined by the number of viable spores resistant to our heat and sonication treatment produced by a given strain in a specified context. '*Absolute fitness*' here simply refers to a strain's sporulation efficiency, i.e. the number of spores produced by a strain relative to the number of vegetative cells of that strain that were initially

subjected to starvation, or D_i (Equation 1) and $D_i(j)$ (Equation 2) for pure and chimeric cultures, respectively. The parameter $C_i(j)$ (Equation 3) quantifies the effect of mixing strains *i* and *j* on the absolute fitness of strain *i,* relative to pure culture conditions, with a positive $C_i(j)$ indicating social exploitation (defined below) of strain *j* by strain *i* and a negative $C_i(j)$ indicating suppression of strain *i* sporulation by strain *j. 'Relative fitness'* here simply refers to W_{ij} (Equation 4), or the relative sporulation efficiencies to two strains in chimeric competition mixes. Defining fitness in terms of spore production under our specific experimental conditions for purposes of this study is not intended to imply anything regarding the role of spore production in the overall fitness of *M. xanthus* strains under natural conditions, which is understood to be determined by a complex array of components, the relative importance of which will vary across environments and over time.

Interaction-specific fitness inequality (ISFI): any inequality in relative fitness between genetically distinct genotypes that is caused specifically by social interactions between those genotypes, irrespective of whether social exploitation (defined below as an interaction-dependent increase in absolute fitness) causes or contributes to the inequality or not.

Non-adaptive or indirect trait evolution: evolutionary trait change that is not caused by natural selection specifically favouring the focal change. This definition encompasses both processes not driven by selection on any trait at all (i.e. mutation and genetic drift), and non-adaptive (at least initially non-adaptive) byproducts of selection caused by pleiotropy or linkage.

Social exploitation: an increase in the absolute fitness of one genotype (e.g. genotype A) specifically caused by social interaction with another genotype (genotype B). Exploitation in this sense may, but need not, cause or contribute to an interaction-specific advantage in relative fitness for genotype A. Buttery et al [1] referred to a negative response of the absolute fitness of one social competitor to interaction with another competitor in the context of *Dictyostelium* development as a form of exploitation by the latter, but following Fiegna and Velicer [2] we apply the label 'exploitation' only to gains in absolute fitness caused by social interaction. Finally, this use of 'exploitation' does not inherently imply a negative effect on the absolute fitness of an exploited strain (even if this may often occur), but rather means simply that the exploiter profits from interaction with another strain in absolute terms, even if the ability do so evolved non-adaptively.

SUPPLEMENTARY TABLES AND FIGURES

Table S1. Evolutionary causation of traits that determine outcomes of competition during a cooperative process. Possible combinations of evolutionary cause (byproduct vs. direct adaptation) and social character (socially fixed vs. responsive) for traits that determine the outcome of competition experiments with microbes engaging in a cooperative process (e.g. fruiting body development).

	**Byproduct competitiveness** – traits determining developmental competition winners/losers evolved as byproducts of something other than selection for developmental competitiveness	**Directly adaptive competitiveness** – traits determining developmental competition winners/losers evolved as adaptations specifically for developmental competitiveness
**Socially fixed** – traits unaltered by the presence of competitors	Socially fixed byproduct	Socially fixed adaptation
**Socially responsive** – traits expressed specifically upon interaction with competitors	Socially responsive byproduct	Socially responsive adaptation

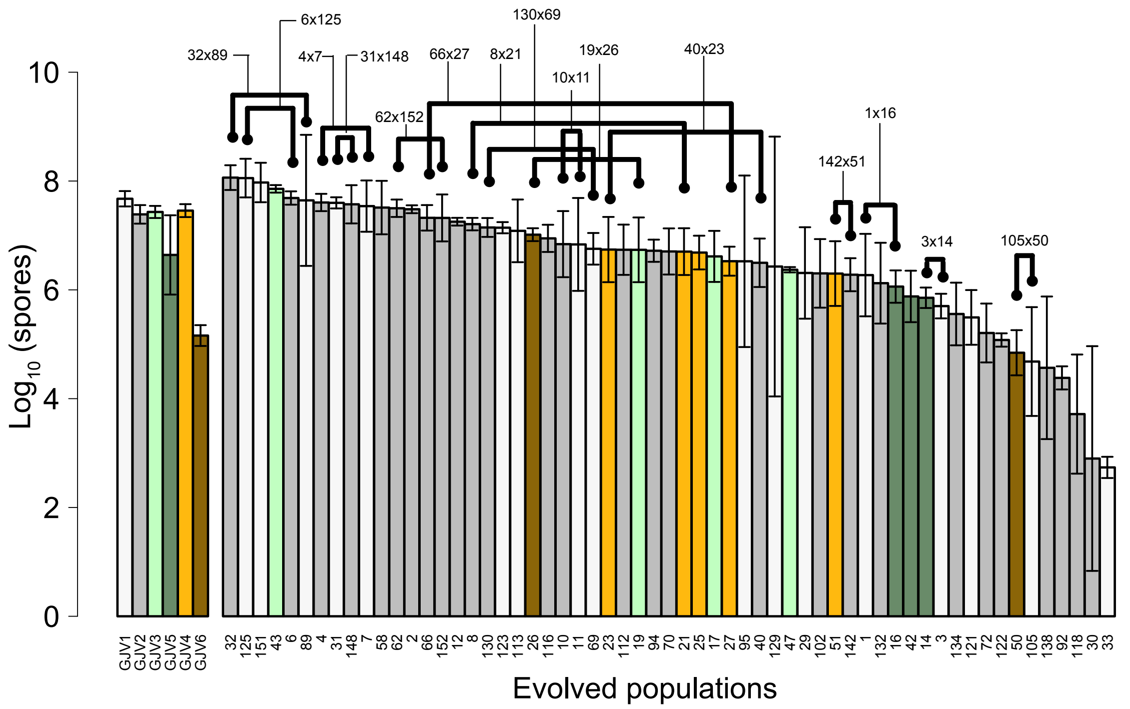

Figure S1. Pure-culture spore production by evolved populations and their ancestors. Colour pairs of white/grey, light/dark green and orange/brown bars denote ancestors (left) with wildtype (A+S+), Δ*cglB* (A-S+) and Δ*pilA* (A+S-) motility genotypes and populations respectively descended from those ancestors (right, Table S3), with the darker colour of each pair indicating rifampicin resistance. Numbers on the x-axis signify the evolved populations (Table S4). Black lines connecting strains show developmental competition pairs. Error bars represent 95% confidence intervals.

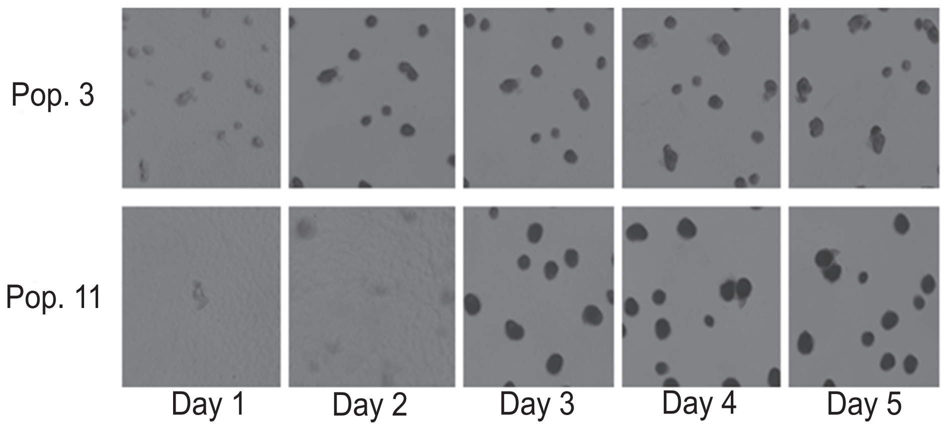

Figure S2. The speed of fruiting-body development diverged among evolved populations. Populations representative of fast developers (darkening of fruiting bodies within two days, Population 3) and slow developers (darkening of fruiting bodies after three days or more, Population 11) are shown.

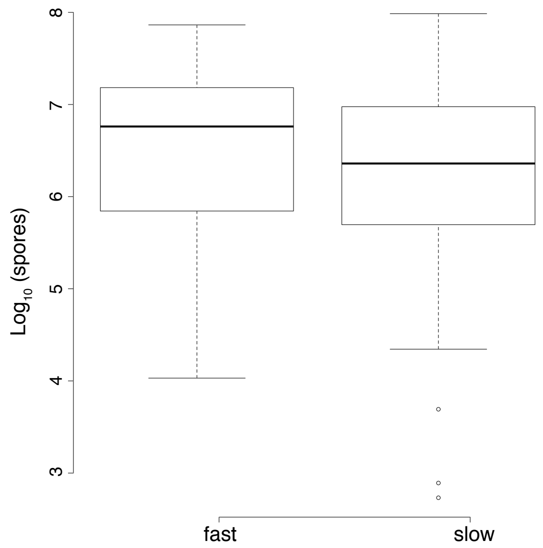

Figure S3. Pure-culture spore production by fast- versus slow-developing evolved strains. The box plots show average sporulation efficiencies from clones isolated from 31 fast-developing and 28 slow -developing populations that exhibited detectable fruiting body formation after 18 cycles of selection for competitiveness at the leading edge of motile swarms undergoing vegetative growth.

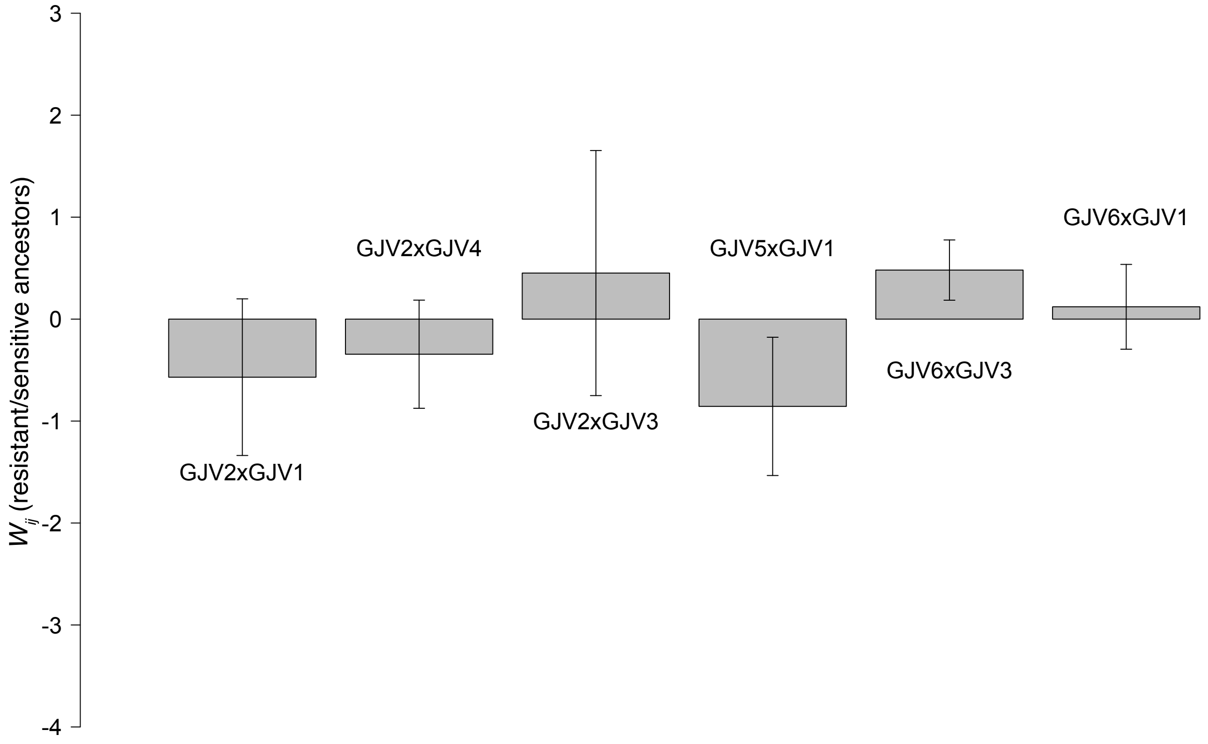

Figure S4. *W_{ij}* values for competitions between relevant ancestral strains. The labels below each bar represent the two competitors, with the rifampicin-resistant competitor (GJV2, GJV5 or GJV6) shown first. Error bars represent 95% confidence intervals.

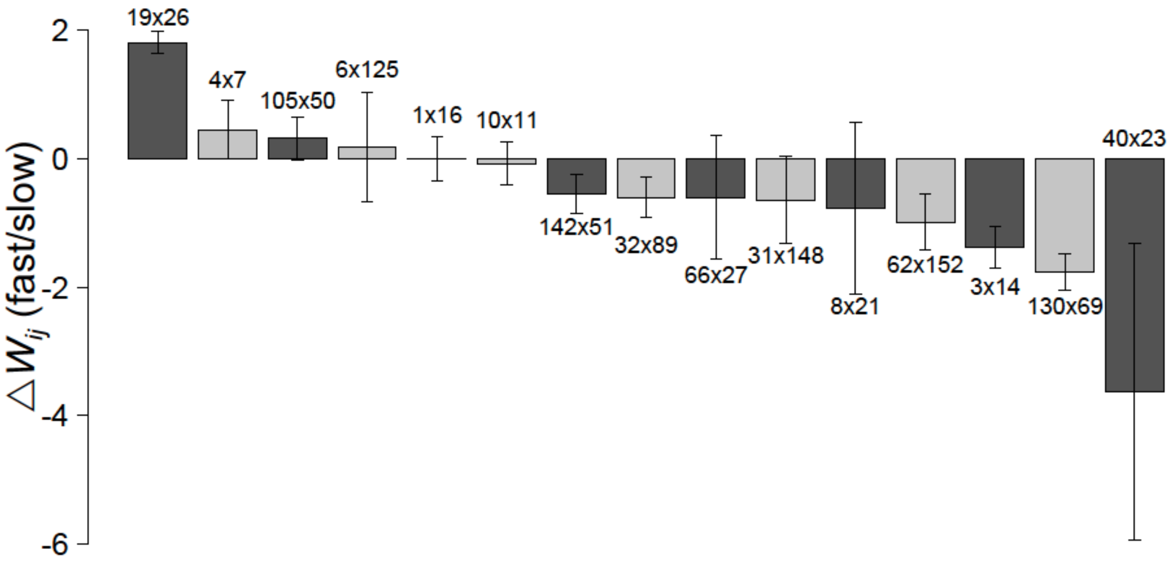

Figure S5. **Estimates of changes in relative fitness specific to experimental evolution.** Each ΔW_{ij} value is an estimate of the divergence in relative developmental fitness between evolved strains during mixed competition assays that occurred specifically during experimental evolution, with estimates of differences in relative fitness of distinct ancestors (Figure S4) factored out (see Methods). For all competition pairs, the strain listed on the left forms fruiting bodies more rapidly than the strain listed on the right. Positive values indicate that the fast-developing strain indirectly increased in relative fitness whereas negative values indicate that the slow-developing strain increased in fitness. Lighter bars denote competitions between strains with identical ancestral motility genotypes. Error bars represent the 95% confidence intervals.

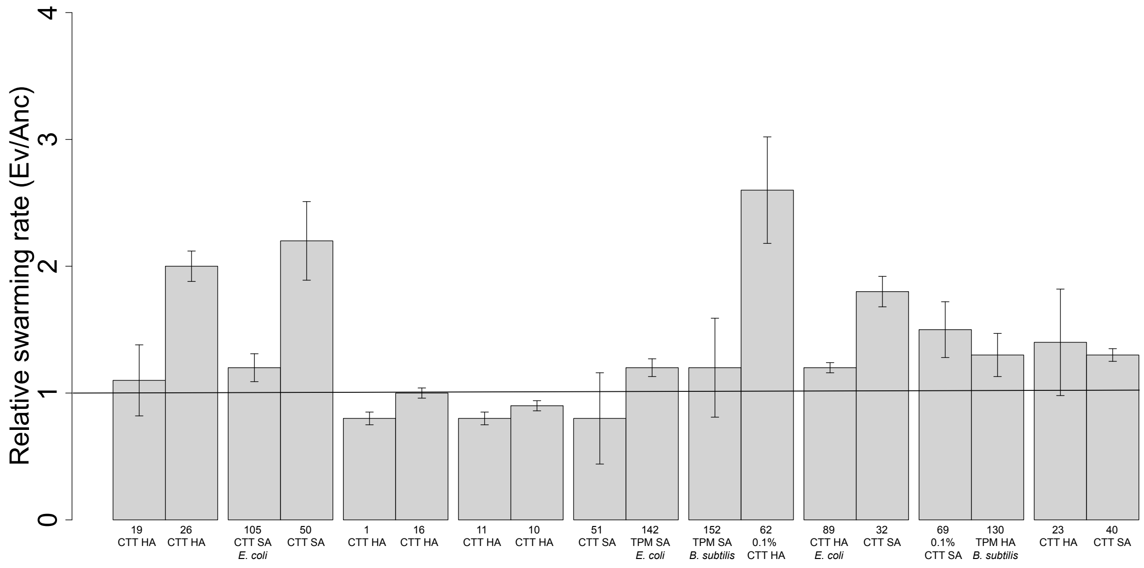

Figure S6. Swarming rate changes are not predictive of performance in developmental competitions. Each value is a ratio of swarming rates by an evolved strain relative to its ancestor (under the selective conditions in which experimental evolution of the focal strain was conducted) with a value of 1 indicating no change. x-axis indicates the respective evolved strains (numbers) and the media used for the respective assay. Competition pairs from Figure 2 are grouped together and follow the same order. Error bars represent 95% confidence intervals.

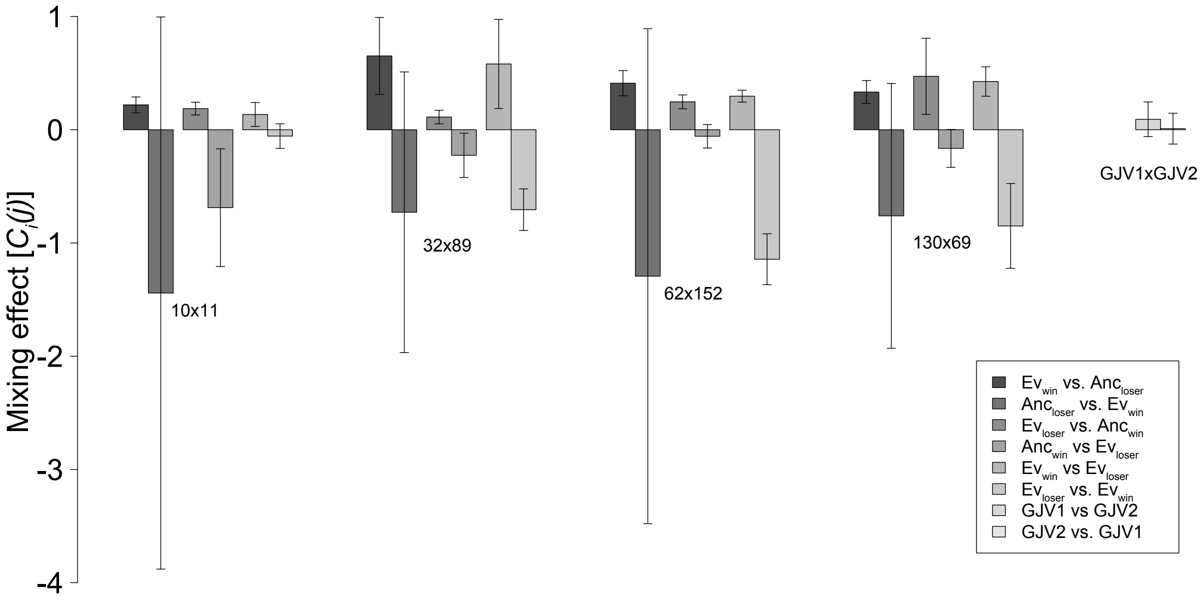

Figure S7. One-way mixing effect for individual competitions involving evolved strains and/or their ancestors. The effect of mixing two clones on the (log-transformed) sporulation efficiency is given as $C_i(j)$ with '*i*' referring to the strain on the left in each pair of values and in the legends and '*j*' referring to the strain on the right. A value of zero indicates no change in absolute spore production of strain '*i*' in mixes compared to its pure culture spore production. Error bars represent 95% confidence intervals.

Table S2. Ancestral strain motility genotypes and marker states.

Ancestor	Motility genotype	Rifampicin marker state
GJV1	A+S+	Sensitive
GJV2	A+S+	Resistant
GJV3	A-S+ (Δ*cglB*)	Sensitive
GJV5	A-S+(Δ*cglB*)	Resistant
GJV4	A+S- (Δ*pilA*)	Sensitive
GJV6	A+S- (Δ*pilA*)	Resistant

Table S3. Summary of experimental-evolution environments.

Ancestors	Evolved populations	Evolution environment
GJV1, GJV2	1 – 12	CTT[a], 1.5% agar
(A+S+)	29 – 40	CTT, 0.5% agar
	57 – 64	0.1% CTT, 1.5% agar
	65 – 72	0.1% CTT, 0.5% agar
	89 – 96	*Escherichia coli* grown on CTT, 1.5% agar
	97 – 104	*Bacillus subtilis* grown on CTT, 1.5% agar
	105 – 112	*E. coli* grown on CTT, 0.5% agar
	113 – 120	*B. subtilis* grown on CTT, 0.5% agar
	121 – 128	*E. coli* overlaid on TPM[b], 1.5% agar
	129 – 136	*B. subtilis* overlaid on TPM, 1.5% agar
	137 – 144	*E. coli* overlaid on TPM, 0.5% agar
	145 – 152	*B. subtilis* overlaid on TPM, 0.5% agar
GJV3, GJV5	13 – 20	CTT, 1.5% agar
(A-S+)	41 – 48	CTT, 0.5% agar
GJV4, GJV6	21 – 28	CTT, 1.5% agar
(A+S-)	49 - 56	CTT, 0.5% agar

a: nutrient rich medium; b: starvation medium

Table S4. Time from initiation of starvation to appearance of darkened fruiting bodies for 59 evolved strains that exhibited fruiting body formation and spore production. Black cells indicate days on which darkened fruiting bodies were observed.

Evolved population	Ancestor	Change in onset of development (days)	Day 1	Day 2	Day 3	Day 4	Day 5
1	GJV1	=		■	■	■	■
2	GJV2	-1	■	■	■	■	■
3	GJV1	-1	■	■	■	■	■
4	GJV2	=		■	■	■	■
6	GJV2	-1	■	■	■	■	■
7	GJV1	+1			■	■	■
8	GJV2	=		■	■	■	■
10	GJV2	=		■	■	■	■
11	GJV1	+1			■	■	■
12	GJV2	-1	■	■	■	■	■
14	GJV5	+2				■	■
16	GJV5	+1			■	■	■
17	GJV3	=		■	■	■	■
19	GJV3	-1	■	■	■	■	■
21	GJV4	+2				■	■
23	GJV4	+2				■	■
25	GJV4	+2				■	■
26	GJV6*	-					■
27	GJV4	+2				■	■
29	GJV1	+3					■
30	GJV2	+3					■
31	GJV1	-1	■	■	■	■	■
32	GJV2	-1	■	■	■	■	■
33	GJV1	+3					■
40	GJV2	-1	■	■	■	■	■
42	GJV5	=		■	■	■	■
43	GJV3	=		■	■	■	■
47	GJV3	=		■	■	■	■
50	GJV6*	-					■
51	GJV4	+1			■	■	■
58	GJV2	+1			■	■	■
62	GJV2	=		■	■	■	■
66	GJV2	-1	■	■	■	■	■
69	GJV1	+1			■	■	■
70	GJV2	-1	■	■	■	■	■
72	GJV2	+1			■	■	■
89	GJV1	+2				■	■
92	GJV2	+1			■	■	■
94	GJV2	+1			■	■	■
95	GJV1	+1			■	■	■
102	GJV2	+1			■	■	■
105	GJV1	-1	■	■	■	■	■
112	GJV2	+3					■
113	GJV1	-1	■	■	■	■	■
116	GJV2	-1	■	■	■	■	■
118	GJV2	+3					■
121	GJV1	=		■	■	■	■
122	GJV2	-1	■	■	■	■	■
123	GJV1	-1	■	■	■	■	■
125	GJV1	+1			■	■	■
129	GJV1	+1			■	■	■
130	GJV2	-1	■	■	■	■	■
132	GJV2	=		■	■	■	■
134	GJV2	=		■	■	■	■
138	GJV2	=		■	■	■	■
142	GJV2	=		■	■	■	■
148	GJV2	+1			■	■	■
151	GJV1	=		■	■	■	■
152	GJV2	+1			■	■	■

*No numerical change in onset of development is given for strains descended from GJV6 because GJV6 itself did not form darkened fruiting bodies, although some of its descendants (populations 26 and 50) did. These populations were counted as having developed earlier than their ancestor (-). All other ancestors (GJV1-5) first produced dark fruiting bodies after two days.

Table S5. Developmental competition pairings and respective ancestors. Competitions between strains evolved from same ancestral motility genotype are highlighted (gray rows).

Fast strain	Ancestor of fast strain	Slow strain	Ancestor of slow strain
19	GJV3	26	GJV6
4	GJV2	7	GJV1
105	GJV1	50	GJV6
6	GJV2	125	GJV1
1	GJV1	16	GJV5
10	GJV2	11	GJV1
142	GJV2	51	GJV4
32	GJV2	89	GJV1
66	GJV2	27	GJV4
31	GJV1	148	GJV2
8	GJV2	21	GJV4
62	GJV2	152*	GJV2
3	GJV1	14	GJV5
130	GJV2	69	GJV1
40	GJV2	23	GJV4

*Population 152 descended from GJV2 but unexpectedly exhibited sensitivity to rifamipicin, suggesting evolutionary loss of resistance. All other evolved populations listed exhibited the same resistance state of their ancestor.

Table S6. Shared vs. different motility genotypes and evolution environments of evolved-population clones paired for developmental competition experiments.

	Evolution environment	
**Motility genotype**	Same	Different
Same	Two pairs<br>*4-vs-7; 10-vs-11*	*Five pairs*<br>*6-vs-125; 130-vs-69; 31-vs-148; 32-vs-89; 62-vs-152;*
Different	Four pairs<br>*1-vs-16; 3-vs-14; 8-vs-21; 19-vs-26*	Four pairs<br>*23-vs-40; 27-vs-66; 50-vs-105; 51-vs-142*

REFERENCES

1. Buttery NJ, Rozen DE, Wolf JB, Thompson CRL. 2009 Quantification of social behavior in D. discoideum reveals complex fixed and facultative strategies. *Curr. Biol.* **19**, 1373–1377. (doi:10.1016/j.cub.2009.06.058)
2. Fiegna F, Velicer GJ. 2005 Exploitative and hierarchical antagonism in a cooperative bacterium. *PLoS Biol.* **3**, e370. (doi:10.1371/journal.pbio.0030370)
